# Treatment of breast cancer in vivo by dual photodynamic and photothermal approaches with the aid of curcumin photosensitizer and magnetic nanoparticles

**DOI:** 10.1038/s41598-020-78241-1

**Published:** 2020-12-03

**Authors:** Ali Ashkbar, Fatemeh Rezaei, Farnoosh Attari, Saboura Ashkevarian

**Affiliations:** 1 Department of Physics, K. N. Toosi University of Technology, Tehran, Iran; 2 Department of Animal Biology, School of Biology, College of Science, University of Tehran, Tehran, Iran; 3 Research Institute of Applied Sciences, ACECR, Shahid Beheshti University, Tehran, Iran; 4 Institute of Biochemistry and Biophysics, University of Tehran, Tehran, Iran

**Keywords:** Biophysics, Cancer, Diseases, Nanoscience and technology, Physics

## Abstract

Breast cancer is a neoplastic disease with a high mortality rate among women. Recently, photodynamic therapy (PDT) and photothermal therapy (PTT) attracted considerable attention because of their minimal invasiveness. The PTT approach works based on hyperthermia generation, and PDT approach employs laser irradiation to activate a reagent named photosensitizer. Therefore, in the current paper, a dual-functioned nanocomposite (NC) was designed for the treatment of breast cancer model in Balb/c mice with the combination of photodynamic and photothermal approaches. Transmission electron microscopy, UV–visible spectroscopy, FTIR, and XRD were employed to validate the nanostructure and silica coating and curcumin (CUR) immobilization on the Fe_3_O_4_ nanoparticles. The effect of Fe_3_O_4_/SiO_2_-CUR combined with PDT and PTT was assessed in vivo on the breast tumor mice model, and immunohistochemistry (IHC) was employed to evaluate the expression of apoptotic Bax and Caspase3 proteins. The TEM images, UV–visible absorption, and FTIR spectra demonstrated the successful immobilization of curcumin molecules on the surface of Fe_3_O_4_/SiO_2_. Also, MTT assay confirmed the nontoxic nature of Fe_3_O_4_/SiO_2_ nanoparticles in vitro. In the breast tumor mice model, we have assessed six treatment groups, including control, CUR + PDT, Blue + NIR (near-infrared) lasers, NC, NC + PTT, and NC + PDT + PTT. The tumor volume in the NC + PDT + PTT group showed a significant reduction compared to other groups (p < 0.05). More interestingly, the tumor volume of NC + PDT + PTT group showed a 27% decrease compared to its initial amount. It should be noted that no detectable weight loss or adverse effects on the vital organs was observed due to the treatments. Additionally, the IHC data represented that the expression of proapoptotic Bax and Caspase3 proteins were significantly higher in the NC + PDT + PTT group compared to the control group, indicative of apoptosis. To conclude, our data supported the fact that the NC + PDT + PTT strategy might hold a promising substitute for chemotherapy for the treatment of triple-negative breast cancers.

## Introduction

Breast cancer is one of the deadliest diseases among women, which leads to metastasis to vital organs such as the lungs and bones in the body. Approximately one in eight women is encountered with breast cancer in her lifetime^1,2^. Recent studies on cancer treatment try to find the best and the least risky way to replace the old methods. The major problem in the currently employed cancer treatments is their adverse side effects on the healthy tissues. Nowadays, various methods are being used for the treatment of breast cancer, such as surgery, targeted therapy, hormone therapy, radiation therapy, chemotherapy, photodynamic therapy (PDT), and photothermal therapy (PTT)^3^. Among these methods, PDT and PTT are valid alternative techniques that recently attracted considerable attention for the treatment of a broad range of diseases, including malignant tumors, because of their minimal invasiveness. The PTT approach is a promising strategy that is based on hyperthermia generation utilizing the light energy (NIR laser) to produce heat in the desired tissue^4^. PDT is a phototherapy approach in which laser irradiation can activate a reagent named photosensitizer (PS) and subsequent production of ROS molecules, which lead to the destruction of the cancerous area^5^. Generally, photosensitizers play a pivotal role in PDT due to their ability in the production of singlet oxygen via light absorption at the peak wavelength of PS^6,7^.

Photo-active nanostructures are ideal diagnostic detection agents for biosensing^8^, drug delivery^9^, and therapy^10^, which provide significant tools in signal enhancement, photodynamic and photothermal therapy^11^, and non-linear optical imaging systems^12^. Moreover, utilizing nanoparticles (NPs) for drug delivery is a growing area in the research which controls the rate of drug release in the desired tissue while circumventing the unwanted delivery to the undesired places^9^. On the other hand, herbal drugs and their derivative phytocompounds are nowadays known as valuable complementary treatment materials for cancer therapy. Recent reports employed phototherapeutic approaches in combination with nanotechnology and phytochemicals in order to achieve suitable nanocomposite materials for targeted drug delivery^13^.

Different research groups have studied the treatment of breast cancer with the simultaneous usage of PDT or PTT and nanoparticles. For instance, Stuchinskaya et al., have used targeted PDT employing conjugates of antibody-phthalocyanine-gold nanoparticles for the treatment of HER2 positive breast cancer cells^14^. Besides, gold nanorods coated with SiO_2_ and loaded with Ce6 photosensitizer, in combination with PDT and PTT, induced a strong cytotoxic effect on MCF-7 breast cancer cells^15^. Furthermore, Beqa et al. have designed a new hybrid nanomaterial based on gold popcorn-attached carbon nanotubes for appropriate diagnosis and targeted photothermal treatment, which produced irreparable damage to cancer cells within 10 min at 1.5 W/cm^2^ laser power^16^. They have shown that the hybrid nanomaterial worked far better compared to the single nanomaterial in response to the photothermal irradiation.

Today, the successful application of nanoparticles in combination with photodynamic and photothermal techniques have illustrated drastic improvements in the therapeutic process^17,18^. Curcumin is an herbal compound with antioxidant and photosensitizer properties, which decreases inflammation and bears anticancer properties^19^. To our knowledge, curcumin was not employed in a nanostructure in combination with PDT and PTT approaches so far, and the mentioned strategy was not studied in vivo on breast cancer, as well. Therefore, in the current study, a dually functioned nanocomposite was designed for the treatment of breast cancer model in Balb/c mice with the simultaneous employment of photothermal and photodynamic approaches. Here, silica-coated Fe_3_O_4_ magnetic nanoparticles which were loaded with curcumin (CUR), as a natural photosensitizer, were injected to the tumor site and CW diode lasers at 450 nm for PDT and at 808 nm for PTT were irradiated on the tumor area for the simultaneous production of hyperthermia and singlet oxygen to improve the treatment process.

## Materials and methods

### Materials

In this study, curcumin was loaded into Fe_3_O_4_-SiO_2_ nanoparticles. Curcumin was supplied from medicinal plants and drugs research Institute of Shahid Beheshti University. Tetraethyl orthosilicate (TEOS) was purchased from sigma-aldrich, ferric chloride hexahydrate (FeCl_3_·6H_2_O), ferrous sulfate heptahydrate (FeSO_4_·7H_2_O), NH_3_·H_2_O, phosphate buffer saline (PBS), acetic acid, ethanol, and methanol were obtained from Merck for the construction of the composite nanoparticle. All chemicals were analytically graded and utilized without any additional purification. The diode laser at 450 nm for PDT and another diode NIR laser at 808 nm for PTT were supplied from Takfamsazanshafa Company. In addition, an external magnetic field was used for proper drug delivery.

### Synthesis of Fe_3_O_4_ nanoparticles

For the synthesis of Fe_3_O_4_ nanoparticles, the chemical co-precipitation method for Fe_3_ and Fe_2_ ions was utilized. First, 3.40 g of FeCl_3_·6H_2_O and 1.25 g of FeSO_4_·7H_2_O were dissolved in 100 mL of deionized water by strong stirring. Then, 6 mL of 25% NH_3_·H_2_O was added to the suspension at 60 °C and was shaken severely for 30–40 min. The solution color promptly changed into black because of the formation of Fe_3_O_4_. The obtained nanoparticles were washed twice with PBS and once with ethanol to be prepared for silica coating^20^.

### Preparation of silica-coated Fe_3_O_4_ nanoparticles

At first, 75 mg of the prepared Fe_3_O_4_ was added to 5 mL toluene and dispersed under ultrasonication for 10 min. Then, this solution was added to a mixture containing 20 mL deionized water, 80 mL ethanol, and 2.5 mL ammonium hydroxide. Next, 1 mL TEOS was added to the suspension drop by drop while being stirred at 25 °C. For separation of the silica-coated nanoparticles, a permanent magnet was used, followed by three-times washing with ethanol and drying in the vacuum for 24 h at 60 °C^20^.

### Immobilization of curcumin on silica-coated Fe_3_O_4_ nanoparticles

For curcumin immobilization, 2 mg of the silica-coated nanoparticles was added to 1 mL of 100 mM sodium phosphate buffer (pH 7.4), containing 2 mg/mL of curcumin while being softly stirred at 25 °C for 24 h. Then, the immobilized curcumin derivatives were collected by a magnet and washed with PBS buffer. The presence of absorbance wavelength at 435 nm in the spectrophotometric analysis was indicative of curcumin immobilization on the nanoparticles.

### Measurements for characterization of the obtained nanocomposite (NC)

A transmission electron microscope imaging system (TEM) (Zeiss-EM10c) operating at 100 kV was utilized to study the morphology, particle size and to evaluate the core–shell structure of silica-coated Fe_3_O_4_ nanoparticles. In addition, the Fourier-transform infrared spectroscopy (FTIR) analysis was performed by spectrophotometer ABB Bomem (model FTLA2000) to prove the immobilization of curcumin on the magnetic Fe_3_O_4_/SiO_2_ nanoparticles. To illustrate the stabilization of curcumin on Fe_3_O_4_/SiO_2_, the FTIR spectroscopy of three conditions: (i) curcumin, (ii) Fe_3_O_4_/SiO_2_, and (iii) Fe_3_O_4_/SiO_2_-CUR nanocomposites were assessed. To show the presence of curcumin on the surface of nanoparticles, the UV/Visible absorbance of pure and immobilized curcumin at 435 nm wavelength was investigated using a Shimadzu UV-160 spectrophotometer. For this purpose, 2 mg/mL of pure curcumin solution (2 mg curcumin/mL PBS) and 2 mg/mL of Fe_3_O_4_/SiO_2_-CUR solution were measured at 435 nm. Magnetic properties of the synthesized Fe_3_O_4_ nanoparticle were determined using a vibrating sample magnetometer (VSM, Mahamax Co., Iran). Zeta potentials and nanoparticle size were also measured using Zeta PALS and Zeta Sizer (Zeta Plus, Brookhaven, USA). The crystalline phases of Fe_3_O_4_ nanoparticles and nanocomposits were identified using a Philips Analytical X-ray diffractometer (XPert MPD).

### Calculation of drug loading

To determine the curcumin loading on the surface of Fe_3_O_4_ NPs, 2 mg of silica-coated NPs and curcumin powder were added separately into 1 mL of PBS and sonicated for 10 min. Then, curcumin solution was added to NP solution and incubated at 4 °C for 24 h. Using a magnet, Fe_3_O_4_/SiO_2_-CUR NCs were washed five times with PBS to remove unstabilized curcumin. The content of curcumin was characterized by UV–Vis spectroscopy at the wavelength of 435 nm. Equation (1) is used to calculate drug loading:

1 $$ {\text{Drug loading }}\left( \% \right) = {\text{ W}}_{0} - {\text{W}}_{{1}} /{\text{W}}_{{{\text{NP}}}} \times {1}00 $$

where W_0_, W_1_, and W_NP_ represent the initial weight of curcumin, the weight of the detected curcumin in the solution, and weight of the Fe_3_O_4_/SiO_2_-CUR, respectively.

### In vitro release of curcumin from the NCs

To determine the drug release properties of the Fe_3_O_4_/SiO_2_-CUR, dialysis bags (cut-off 12 kDa), containing 1 mg/mL of pure curcumin or Fe_3_O_4_/SiO_2_-CUR were immersed in vessels containing 10 mL PBS (pH 7.4) and incubated at 37 °C on a shaker (100 rpm). This procedure was done in the absence and presence of irradiation (3 min). At determined intervals, 1 mL of the released solution was replaced with the same amount of fresh PBS, and the release profile was analyzed by a UV–Vis spectrophotometer at 435 nm. The release percentage of curcumin was calculated using Eqs. (2) and (3)^21^.

2 $$ {\text{W}}_{{{\text{rmod}}}} = {\text{ W}}_{{\text{r}}} + \, \left( {{\text{V}}_{{\text{s}}} /{\text{V}}_{{\text{t}}} } \right){\text{SW}}_{{\text{r}}} $$

3 $$ {\text{Release rate }}\left( \% \right) \, = {\text{ W}}_{{{\text{rmod}}}} /{\text{W}}_{{{\text{total}}}} \times {1}00 $$

where W_r_ and W_rmod_ represent the evident weight and modified weight at time t, respectively, V_S_ was the volume of the taken sample, V_t_ was the total volume of the released medium, ∑W_r_ was the summation of W_r_, and W_total_ was the total weight of curcumin present on the surface of nanoparticles.

### Colloidal stability

Colloidal stability analysis was conducted at 25 °C for seven days using DLS. Samples were prepared in deionized water with the adjusted concentrations at 0.46 mg/mL. Equation (4) was used to determine the colloidal stability of the particles^21^.

4 $$\text{Colloidal Stability} \; \text{t}_\text{n} =\frac{\text{Nano carrier size }(\text{t}_\text{n})}{\text{Initial size of nano carriers }(\text{t}_{0})}$$

where the colloidal stability of the particles in each day (t_n_) equaled the nanocarrier size in each day (t_n_) to the initial size of the nanocarrier at the beginning of the test (t_0_).

### Toxicity evaluation by MTT assay

To assess toxicity of Fe_3_O_4_ nanoparticles and NCs in vitro, MTT assay was employed as reported previously^22^. In summary, 4T1 cells were cultured in 96-well plates with a density of 10^4^ cells/well overnight. Then, cells were treated with different concentrations of Fe_3_O_4_ nanoparticles or NC for 24 and 48 h. At the end of treatment time, MTT solution (Sigma-Aldrich, USA) with the final concentration of 0.5 mg/ml was added to each well and incubated for 4 h at 37 °C. Next, the MTT containing medium was removed, and 100 μL DMSO was added to solve the formazan crystals. The absorbance of purple color was determined using a microplate Eliza reader (BioLegend, USA).

### In vivo experiment

Female Balb/c mice (6 to 8 weeks) were provided by Tehran University of Medical Sciences; all the experiment protocols were approved by Tehran University of Medical Sciences and University of Tehran (School of Biology, 990604368) licensing committee. All methods were carried out in accordance with relevant guidelines and regulations. The mice were injected with 1 × 10^6^ 4T1 cells subcutaneously, and the treatment started when the tumor size reached about ~ 100 mm^3^. The tumorized mice were divided into six groups of ≥ four replicates, which received: (I) 40 µL of PBS injection (control group), (II) injection of 40 µL PBS containing 100 µg curcumin plus irradiation with a blue diode laser at 450 nm with the intensity of 150 mW/cm^2^ for 3 min (CUR + PDT group), (III) PBS injection plus irradiations with blue diode laser for 3 min followed by NIR laser with the intensity of 0.5 W/cm^2^ for 7 min (Blue + NIR lasers group), (IV) injection of 40 µL nanocomposite (NC group), (V) injection of 40 µL of NC solution containing 20 µg curcumin (0.46 mg/mL) plus irradiation with NIR laser at 808 nm for 7 min (NC + PTT), and (VI) injection of 40 µL of nanocomposite containing 20 µg curcumin plus irradiations with two lasers with the mentioned intensity and exposure times while a rigid magnet was fixed on the tumor to maintain the injected nanocomposite in the tumor position (NC + PDT + PTT group). The injections were performed intratumorally, and the mentioned treatment procedures were performed every other day for 2 weeks. The tumor size was measured by a caliper with 0.1 mm resolution, and tumor volume was calculated with the following formula: V = pi/6 × (L × W × W); L as the length and W as the width of the tumor. The tumor temperature during the irradiations was measured by an Infrared thermometer (Benetech).

### Immunohistochemistry analysis

The tumors were fixed in 4% paraformaldehyde, embedded in paraffin, and sectioned into 3 µm slices. After the deparaffinizing process, sections were incubated in citrate buffer for 20 min in 70 °C, and retrieval was performed with 2 M HCL for 30 min. Next, 0.3% Triton was used for cell permeability, and blocking was done with 10% goat serum. For the next step, mouse monoclonal anti-Bax or anti-Caspase3 antibody (1:100 dilution, Dako) was added to the slides and incubated overnight at 4 °C followed by secondary antibody incubation in the next day and DAB reagent treatment (1:50 dilution in the buffer, Dako). In the end, counterstaining with hematoxylin was done, and the slides were analyzed by an expert pathologist.

### Statistical analysis

All data were shown as mean ± SEM, and comparison between the groups was performed by one-way ANOVA analysis and the post hoc test in GraphPad Prism 5, with p < 0.05 considered statistically significant.

## Results

### Characterization of nanocomposites

**Figure 1:**
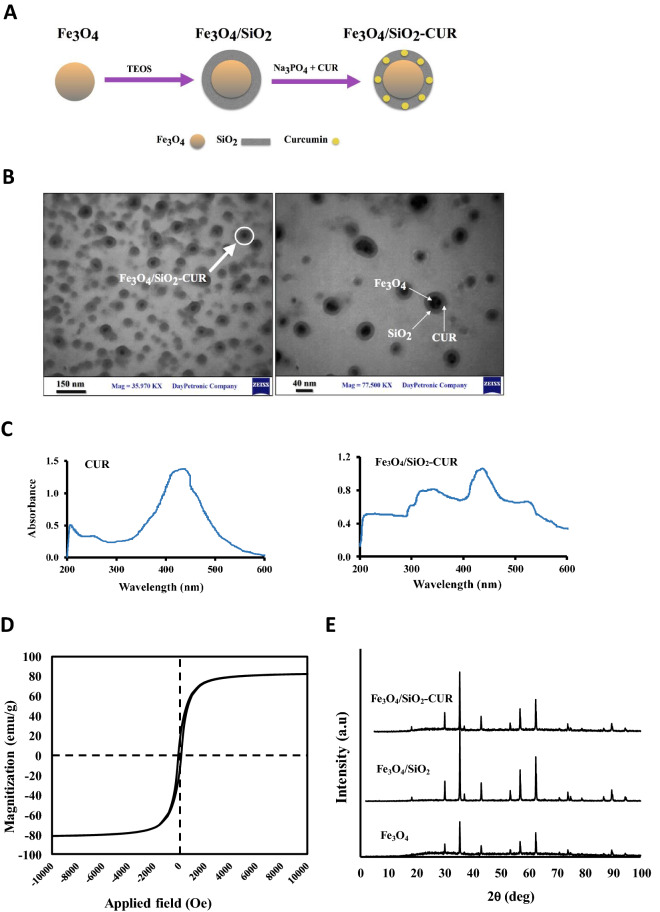
(**A**) Schematic representation of the structure of Fe_3_O_4_/SiO_2_-CUR nanocomposite containing the Fe_3_O_4_ core and a silica shell with curcumin molecules. (**B**) Transmission electron microscopy (TEM) images of the nanocomposite. Dots with 10–30 nm diameters represent Fe_3_O_4_ nanoparticles, the colorless borders around show SiO_2_ coating, and dots < 5 nm are curcumin loaded in SiO_2_. (**C**) The UV–visible absorption spectrum of curcumin (CUR), and Fe_3_O_4_/SiO_2_-CUR nanocomposite. (**D**) VSM Magnetic measurements for synthesized Fe_3_O_4_ magnetite nanoparticles with the applied magnetic field sweeping from − 10 KOe to 10 KOe. (**E**) XRD patterns of naked Fe_3_O_4_, Fe_3_O_4_/SiO_2,_ and Fe_3_O_4_/SiO_2_-CUR nanocomposite using an X-ray diffractometer.

The TEM analysis used for investigating the size and formation of core/shell nanostructure showed that Fe_3_O_4_ nanoparticles were successfully coated with silica to form a core/shell nanostructure (20–60 nm) in which Fe_3_O_4_ and silica could be identified with a dark black dot in the center and a gray halo surrounding the dots, respectively (Fig. 1A,B). The UV–visible absorption spectrum of free curcumin and synthesized nanocomposite (Fig. 1C) showed the maximum absorption at 435 nm for the pure and immobilized curcumin, which confirmed the successful loading of curcumin molecules on the surface of Fe_3_O_4_/SiO_2_ NPs.

To evaluate the magnetic properties of synthesized Fe_3_O_4_ nanoparticles, VSM (vibrating sample magnetometer) analysis was used. As can be seen in Fig. 1D, with the applied magnetic field sweeping from − 10 KOe to 10 KOe, magnetic hysteresis curve showed an S-like shape, indicating the superparamagnetic nature of the NPs and the magnetic response of NPs was 80 emu g^−1^. Moreover, the XRD diffraction patterns of Fe_3_O_4_, Fe_3_O_4_/SiO_2_, and Fe_3_O_4_/SiO_2_-CUR were with single magnetite phases^23^ (Fig. 1E). The presence of the same peaks in the XRD patterns of Fe_3_O_4_/SiO_2_ and Fe_3_O_4_/SiO_2_-CUR proved that silica coating and immobilization of curcumin did not change the magnetite phase of the nanoparticles.

**Figure 2:**
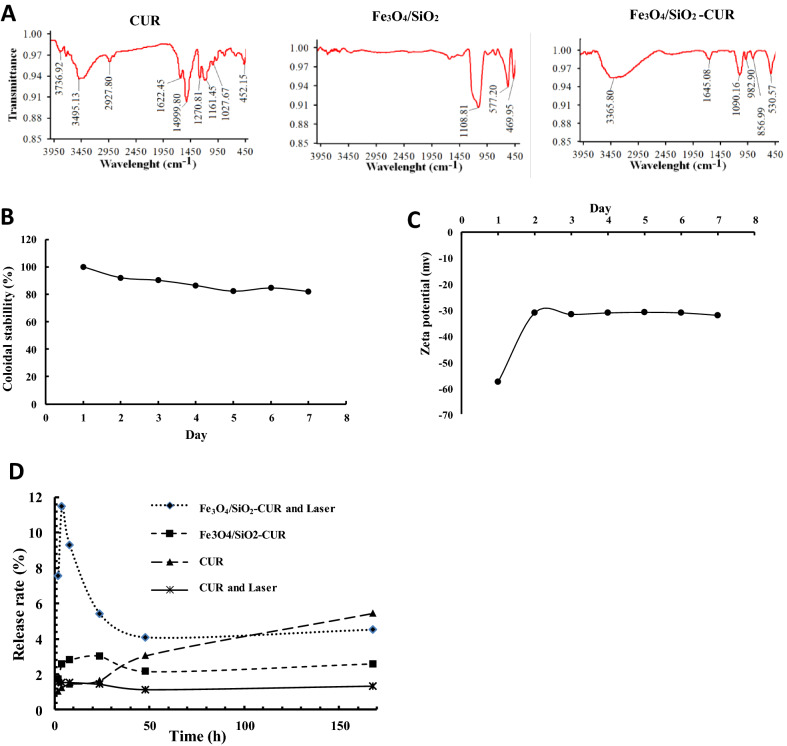
(**A**) FTIR spectra of curcumin (CUR), Fe_3_O_4_/SiO_2_ NP, and Fe_3_O_4_/SiO_2_-CUR nanocomposite. (**B**) Colloidal and (**C**) Zeta potential stability of Fe_3_O_4_/SiO_2_-CUR nanocomposite at pH 7.4 at 25 °C for seven days using DLS technique (**D**) The release profile of CUR and Fe_3_O_4_/SiO_2_-CUR in 168 h without or with laser irradiation (3 min) assessed by UV–Vis spectrophotometer at 435 nm.

In the FTIR graphs, the peak at 1090.16 cm^−1^ was attributed to the symmetric stretching of Si–O–Si bonds in the Fe_3_O_4_/SiO_2_-CUR. This indicated the successful coating of silica shell on the Fe_3_O_4_ nanoparticles. As can be seen in Fig. 2A, there are two new peaks at 556.9 and 982.9 cm^−1^ in the curve of Fe_3_O_4_/SiO_2_-CUR compared to that of CUR, indicating that curcumin was immobilized onto Fe_3_O_4_/SiO_2_ nanoparticles. SiO_2_ exhibited one typical peak at 3745 cm^−1^ assigned to isolated silanols (n(O–H)), whereas curcumin displayed a sharp peak at 3495.1 cm^−1^ and a broad peak at 3000–3550 cm^−1^ indicating OH group vibrations without and with intermolecular hydrogen bonding, respectively. The above three peaks could be observed in the spectrum of Fe_3_O_4_/SiO_2_-CUR nanocomposite. Moreover, a new broad peak centered at 3365.8 cm^−1^ could be seen in Fe_3_O_4_/SiO_2_-CUR NC due to intermolecular hydrogen bonding between isolated silanol and enolic hydroxyl groups. As a result, FTIR spectra confirmed the successful silica coating and immobilization of curcumin on the Fe_3_O_4_ nanoparticles.

Measurement of zeta potential (ζ) was performed to determine the nanoparticles' surface charge. The zeta potentials of Fe_3_O_4_ and Fe_3_O_4_/SiO_2_-CUR were − 18.46 and − 57.5 mV, respectively (Table 1). The reducing trend in zeta potential values of Fe_3_O_4_/SiO_2_ and Fe_3_O_4_/SiO_2_-CUR compared to Fe_3_O_4_ indicated the successful silica coating and curcumin loading on the surface of Fe_3_O_4_ nanoparticles. These results showed that immobilization of curcumin on Fe_3_O_4_ affected the NPs surface charge.

**Table 1:** Zeta potential (ζ) measurements.

	Fe_3_O_4_	Fe_3_O_4_/SiO_2_	Fe_3_O_4_/SiO_2_-CUR	Curcumin
Zeta potential (mV)	− 18.46	− 36.31	− 57.5	− 56.18

### Colloidal stability and drug loading/release

Dynamic light scattering (DLS) was utilized to determine the size (hydrodynamic diameter) and surface charge of nanoparticles and consequently to specify the colloidal stability of nanocarriers. The results demonstrated that the average hydrodynamic diameter of Fe_3_O_4_/SiO_2_-CUR did not change after seven days, and their colloidal stability was 82% (Fig. 2B), indicative of strong fabrication of Fe_3_O_4_/SiO_2_-CUR. Our data also showed that zeta potential of Fe_3_O_4_/SiO_2_-CUR increased after one day due to the detachment of unreacted curcumin caused by sonication; however, this value remained above − 30 mV in the next six days, implying the proper stability of the NCs (Fig. 2C). Nanoparticles with zeta potential values greater than + 25 mV or less than − 25 mV show high degrees of stability, since Van der Waal interactions between particles with low zeta potentials lead to their aggregation^24^. The zeta potential value of the Fe_3_O_4_ NPs was − 15 mV; however, this value and the consequent stability increased after silica coating and curcumin loading.

Based on Eq. (1), the calculated curcumin loading on Fe_3_O_4_/SiO_2_-CUR NC was about 23%, (0.46 mg/mL). Loading efficiency greatly depends on the interactions between the SiO_2_ and curcumin^25,26^. The drug release profile of the Fe_3_O_4_/SiO_2_-CUR in the absence and presence of NIR was compared with free curcumin (pH = 7.4). As shown in Fig. 2D, the release rate of curcumin from Fe_3_O_4_/SiO_2_-CUR was significantly slower than free curcumin. Moreover, in the absence of the laser, the amount of released curcumin from the Fe_3_O_4_/SiO_2_-CUR was 3% in 10 h. However, in the presence of laser, a burst release (11.5%) was observed in the first 10 h followed by a sustained, controlled release over 168 h.

### Dark toxicity effect of Fe_3_O_4_/SiO_2_ nanoparticles and NC on 4T1 cells

**Figure 3:**
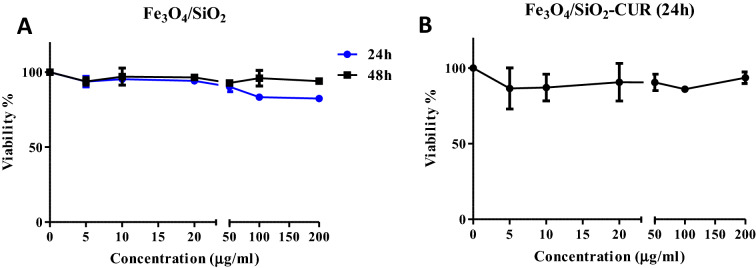
Toxicity of Fe_3_O_4_/SiO_2_ nanoparticles and Fe_3_O_4_/SiO_2_-CUR NC on 4T1 cells in the dark assessed by MTT assay. (**A**) In vitro toxicity of Fe_3_O_4_/SiO_2_ NP after 24 and 48 h. (**B**) In vitro toxicity of Fe_3_O_4_/SiO_2_-CUR NC after 24 h.

To assess the toxicity of Fe_3_O_4_/SiO_2_ nanocarriers in vitro, 4T1 cells were subjected to different concentrations of this material (0, 5, 10, 20, 50, 100, and 200 µg/ml) for 24 and 48 h. As can be seen in Fig. 3A, no significant decrease in cell viability could be detected in either of the treatments. Moreover, NC treatment (containing 1.25, 2.5, 5, 12.5, 25, and 50 µg/ml of curcumin) in the dark did not reduce the cell viability more than 80% in 4T1 cells (Fig. 3B).

### Antitumor effect of nanocomposite plus PDT and PTT approach in vivo

**Figure 4:**
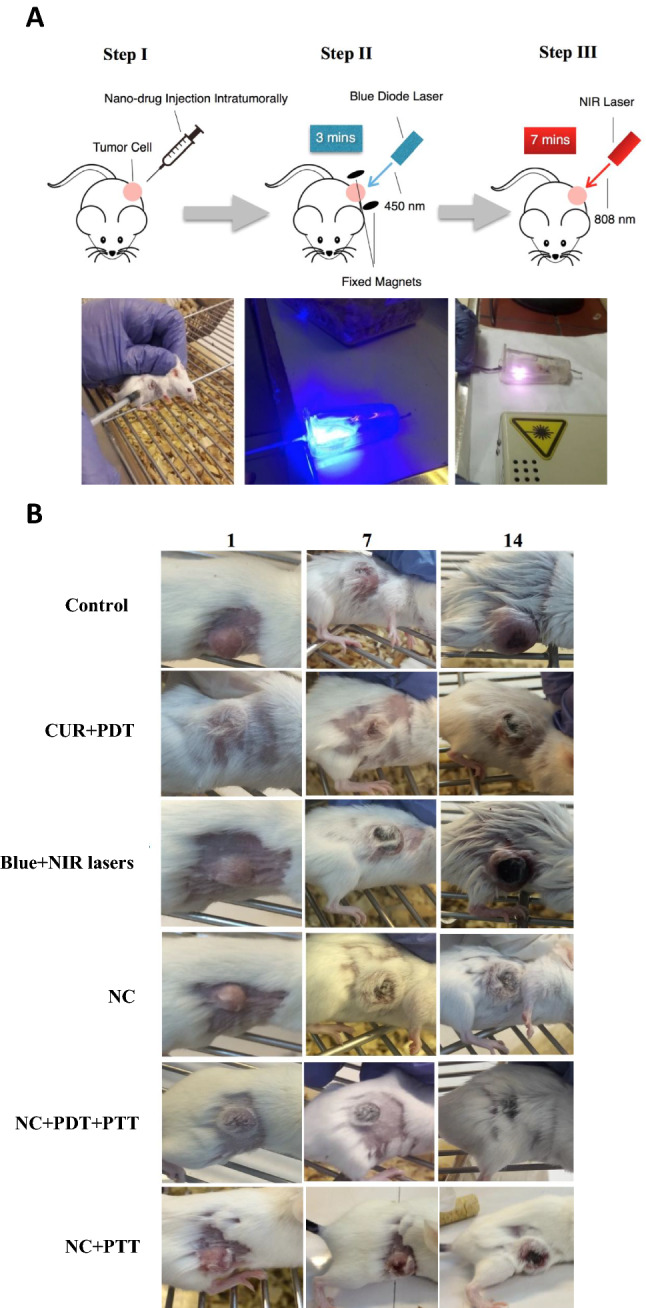
(**A**) A schematic and real demonstrations of treatment steps in the NC + PDT + PTT group which is NC injection (40 µL NC solution composed of 20 µg curcumin and 80 µg Fe_3_O_4_/SiO_2_ nanoparticle) intratumorally followed by blue diode laser irradiation for 3 min and NIR laser irradiation for 7 min. (**B**) The tumor sizes of six groups on days 1, 7, and 14 of treatment.

**Figure 5:**
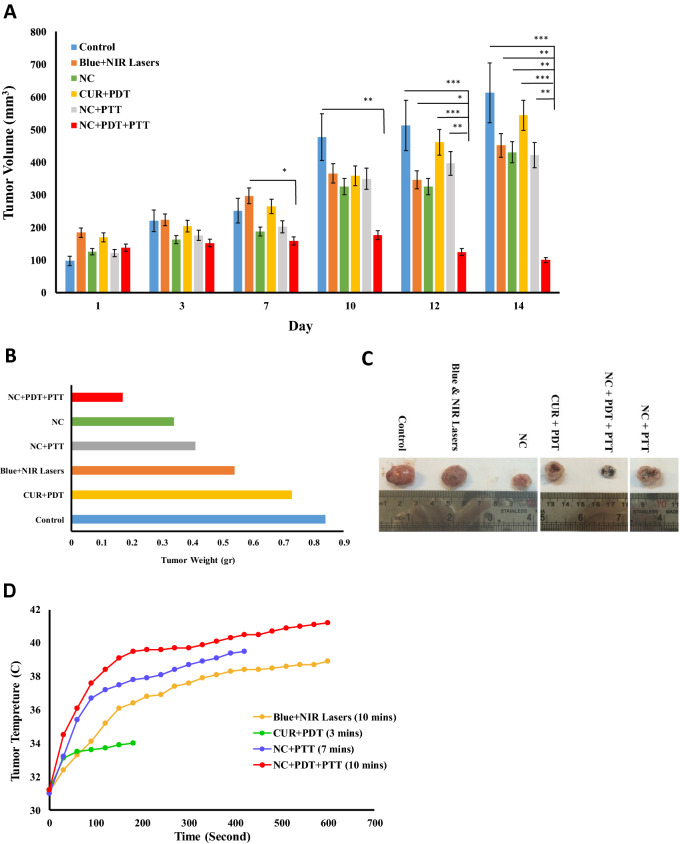
(**A**) Average tumor volume for different treatment groups over 2 weeks. (**B**) Average tumor weights of six treatment groups on day 14. (**C**) The size of harvested tumors from all treatment groups on day 14. (**D**) Changes in tumor temperature during irradiation in Blue + NIR lasers, CUR + PDT, NC + PTT, and NC + PDT + PTT treatment groups employing a thermometer. * p < 0.05, ** p < 0.01, and *** p < 0.001.

**Figure 6:**
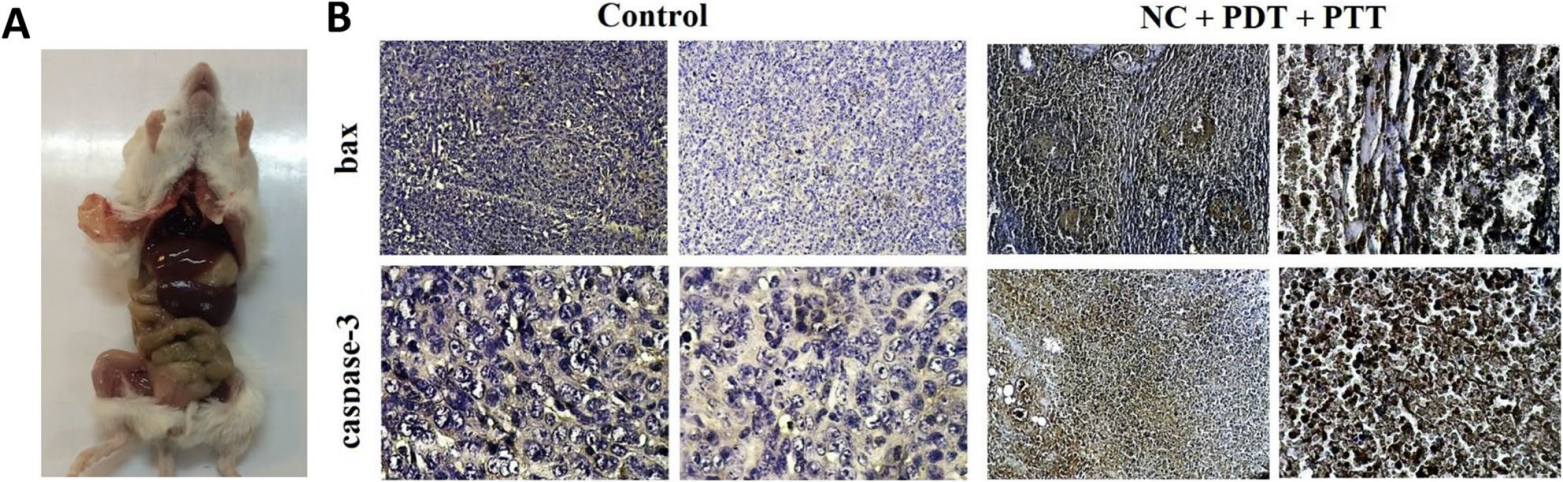
(**A**) Image of inner organs of mice after autopsy related to NC + PDT + PTT group. (**B**) Immunohistochemistry results for the Bax and Caspase3 proapoptotic markers in the control and NC + PDT + PTT groups.

In the current study, due to the observed peak wavelength of the Fe_3_O_4_/SiO_2_-CUR nanocomposites at approximately 450 nm, a blue continuous diode laser at this wavelength seemed to be the best selection for PDT. Moreover, to enhance the treatment process, a near-infrared (NIR) diode laser at 808 nm was used for photothermal therapy (PTT). In the breast tumor mice model, six treatment groups have been assessed, including control, CUR + PDT, Blue + NIR lasers, NC, NC + PTT, and NC + PDT + PTT in which the tumor growth and size were monitored and measured every other day. The treatment procedure of the NC + PDT + PTT group is depicted in Fig. 4A. The size of the tumor in each group on days 1, 7, and 14 of the treatment period is illustrated in Fig. 4B. The tumor volume in the NC + PDT + PTT group showed 94%, 88%, 89%, 87%, and 80% reduction compared to the control, NC + PTT, NC, CUR + PDT, and Blue + NIR lasers groups, respectively (Fig. 5A). More interestingly, the tumor volume in this group on last day of treatment was even less than its primary amount, which means 27% decrease was demonstrated compared to its initial volume. Additionally, the weight of the harvested tumors in this group showed a dramatic reduction in comparison to the other ones, in which 80%, 76%, 68%, 58%, and 50% reduction was observed compared to the control, CUR + PDT, Blue + NIR lasers, NC + PTT, and NC groups, respectively (Fig. 5B). The tumor size of the representatives of each group on day 14 is illustrated in Fig. 5C, in which the NC + PDT + PTT treatment group exhibits the smallest size among all. Tumor temperature in Blue + NIR lasers, CUR + PDT, NC + PTT, and NC + PDT + PTT groups was monitored during irradiation. As can be seen in Fig. 5D, the tumor temperature of NC + PDT + PTT group increased to 41.2 °C after 10 min irradiation. It should be noted that the body weighs of all animal groups were measured on the first and the last day of treatment and no detectable weight loss was observed due to the treatments (data not shown). Also, no sign of adverse effects due to any of the treatments was detected in the vital organs such as the liver and lungs (Fig. 6A).

### Analysis of expression of apoptotic proteins

Since the in vivo experiments demonstrated that the best results belonged to NC + PDT + PTT group, for the next step, the expression of some apoptotic proteins, including Bax and Caspase3 in the control and NC + PDT + PTT groups have been analyzed via immunohistochemistry assay. The IHC data demonstrated that the expression of proapoptotic Bax and Caspase3 proteins were significantly higher in the treated group compared to the control, indicative of apoptosis induction in this group (Fig. 6B).

## Discussion

Due to the limitations of using PDT or PTT alone for cancer treatment, a combination of these approaches with the simultaneous employment of nanoparticles and phytochemicals was considered a proper solution. For that matter, in the present study, we have immobilized curcumin as a photosensitizer reagent on the Fe_3_O_4_/SiO_2_ nanocarriers for the treatment of breast cancer in vivo. The data of TEM analysis, UV–visible absorption, FTIR, and XRD spectra confirmed the successful silica coating and immobilization of curcumin on the Fe_3_O_4_ nanoparticles. Moreover, DLS and zeta potential data indicated the stability of Fe_3_O_4_/SiO_2_-CUR NCs in vitro. The release profile of NC in the presence of laser showed a burst release followed by a sustained, controlled release. Also, the absence of 435 nm peak in the irradiated curcumin could be attributed to its photobleaching^27^. However, the burst release from Fe_3_O_4_/SiO_2_-CUR in the presence of laser could be due to the protective role of nanoparticle for curcumin against laser. Also, the unreleased drug from NC could be related to intermolecular hydrogen bonding of curcumin with silica, which blocked the drug release^28^. The data related to Fe_3_O_4_/SiO_2_-CUR characteristics and release profile suggested that this NC was a proper option to be employed for targeted drug delivery.

To assess the toxicity of Fe_3_O_4_/SiO_2_ nanoparticles we employed MTT assay. The results indicated that Fe_3_O_4_/SiO_2_ nanoparticles used in this study did not exert toxic effects on 4T1 cells. In line with this, different studies reported the non-toxic effect of Fe_3_O_4_ nanoparticles. As an example, viabilities of HeLa and C6 cell lines were not changed due to treatment with PEG-Fe_3_O_4_ NPs even at the concentration of 1 mg/ml^29^. Moreover, another study reported that 24 and 48 h treatment of MCF-7 cells with Fe_3_O_4_ NPs did not alter cell viability, indicating their high biocompatibility^30^. In addition, the manufactured NC in this study did not change in vitro cell viability after 24 h in the dark. This indicates that NC should be triggered and irradiated to release the curcumin, which agrees with the release profile of NC in the dark in which no significant curcumin release was detected in 24 h; however, irradiation triggered curcumin release followed by a delayed release of curcumin over time. This is in agreement with the previous report in which Fe_3_O_4_/ICG@PLGA/PFP nanosystem did not change the viability of MCF-7 cells in the absence of laser irradiation; however, phothothermal toxicity was achieved with this nanosystem in the presence of irradiation^31^. Moreover, curcumin could be entrapped in the pores of the silica coating of NC and needed to be irradiated to get freed.

For in vivo analysis, we have assessed five treatment groups, including CUR + PDT, Blue + NIR lasers, NC, NC + PTT, and NC + PDT + PTT. In our main treatment group (NC + PDT + PTT), curcumin functioned as the PS, which could be activated by the blue laser and produce singlet oxygen, followed by NIR irradiation, which affected Fe_3_O_4_ NPs to generate hyperthermia. The NC was injected into the breast cancer mice model, followed by orderly irradiation with Blue and NIR lasers. It is known that the combination of PDT and PTT approaches requires two different reagents, which would complex the treatment process especially in clinics. That is another reason that we have combined these two reagents into one nanocomposite structure in this study. The in vivo results indicated that the implanted tumors in the control group had a progressive growth during the whole treatment period in which the average tumor volume reached 600 mm^3^ at the end of the treatment that is 530% growth compared to its initial volume. This means that the breast tumor can reach a massive size compared to its primary dimension if no treatment was utilized.

Our data exhibited that CUR + PDT and also NC treatment resulted in about 58% reduction in the tumor volume in comparison with the untreated group. The effect of NC on the tumor volume could be related to the anti-inflammatory and apoptotic properties of curcumin. In the PDT approach, the excitation of inactive photosensitizers by the appropriate wavelength light leads to ROS production and the subsequent apoptosis of tumor cells. Curcumin is a polyphenol and many lines of evidence exist in favor of its chemoprevention and anticancer properties. It is also reported that this reagent can work more effectively if used as a photosensitizer in PDT approach, which led to the more efficient eradication of glioblastoma cells^32^. The effectiveness of curcumin as a PS combined with PDT was proved on prostate cancer treatment, as well^19^. Yet, curcumin possesses a low water solubility, which can be improved by its fabrication with nanoparticles^33^. These data match the observed results in the present study in which loaded curcumin onto Fe_3_O_4_ NPs dissolved more easily in water compared with free curcumin. One of the shortcomings of PDT is its oxygen-consuming feature, which gradually would lead to reduced ROS production and its related apoptosis^34^. Moreover, PDT mostly demonstrates low efficacy since cancer cells require proper uptake of PS. In this regard, some studies reported the enhanced membrane permeability and cellular uptake of some anticancer agents due to a mild photothermal heat (~ 43℃), which can be addressed employing PTT. That is why, in a synergistic attitude, the combination of PTT and PDT strategy can promote cancer treatments^35^. This further supports the idea of employing PDT plus PTT in this study in which, as the PTT reagent, Fe_3_O_4_ iron oxide (IONs) nanoparticles have been utilized. Combining the magnetic field and NIR laser can boost the thermal potential of iron oxide NPs^36^. We have demonstrated increment in the tumor temperature via treatment with NC + PDT + PTT approach, which confirms the previous reports^37^. Moreover, magnetic nanoparticles could be directed toward the tumor area using magnetic fields. Since we have used the intratumoral injection of NC in our study, the magnetic field helped us to maintain NC in the tumor area during the irradiation. It is shown formerly that IONs could be exploited solely as photothermal agents and had the ability to generate hyperthermia under NIR irradiation. A good point in employing iron oxides is their approved usage for resonance imaging in humans. Moreover, IONs have a suitable biodegradability in the body, which makes them good candidates to be used for clinical purposes. In the IONs-mediated hyperthermia, these NPs could be delivered to the tumor site directly, followed by the local heat production and thermal destruction of the tumor. As an example, in a thermotherapy approach, about 66 patients with glioblastoma tumor received iron oxide nanoparticles intratumorally, which led to the longer survival rate of the patients compared to conventional therapies for the treatment of recurrent glioblastoma^38^. When lung tumor-bearing mice received intratumoral IONs followed by irradiation with 808 nm laser (5 W/cm^2^ for 180 s), an obvious volume reduction was observed in the treated tumors^39^.

Additionally, it was reported that the anticancer efficacy of Doxorubicin in ION nanocarriers in combination with PTT (808 nm, 1.5 W/cm^2^ for 3 min) in the tumorized mice has been increased^30^; however, for IONs to exert an efficient hyperthermic property, a high density of irradiation is necessary (over 1 W/cm^2^), yet the safety limit for skin-related tissues is 0.33 W/cm^2^ for 808 nm laser^40^. Interestingly, in the dual PDT plus PTT approach, we could reduce the intensity required for the PTT purpose to 0.5 W/cm^2^, which is approximately near to the safe limit needed for skin-associated tissues. Additionally, in the current study, the combination of PDT and PTT approaches has been assessed to increase the efficiency of anticancer properties of curcumin and IONs.

In the present study, the tumor volume in the NC + PDT + PTT group showed more than 80% reduction compared to the other treatment groups. Furthermore, it should be stressed that this group was the only treatment approach in which the tumor volume reached a magnitude less than its initial volume (27% reduction on the last day compared to the first day of treatment). This significant reduction in the tumor volume could be explained by the produced hyperthermia plus ROS generation in the tumor location. Our findings are in agreement with recent reports on the efficacy of the combination of PDT and PTT in the field of cancer therapy; for instance, PEGylated graphene has been designed as the carrier for the PS chlorin which the obtained composite had a proper water solubility to be used in PDT plus PTT with a better efficacy compared to the free chlorin. Most importantly, the photothermal function of graphene could help the delivery of the PS to the tumor cells, as well^41^. Also, graphene oxide NPs were employed to carry ZnPC as PS, and the remarkable synergistic effect of PDT plus PDT was observed compared to each approach being used alone^42^. Additionally, another report with PDT plus PTT approach showed that Au NPs could produce singlet O_2_ and hyperthermia whose therapeutic potency was observed on cSCC cancer model in vivo^43^. Our data depicted that the main treatment strategy, NC + PDT + PTT, could effectively stop the tumor growth until day ten, followed by the tumor shrinkage till the last day; however, in other treatment strategies, a significant increasing trend in the tumor volume was noticeable.

Interestingly, the immunohistochemistry results revealed that the expression of apoptotic factors, including Bax and Caspase3 increased significantly in the NC + PDT + PTT group compared to the control group. It confirms the fact that the tumor shrinkage in this group could be associated with apoptosis occurrence via the mitochondria pathway. In line with our results, it was demonstrated that under PDT plus PTT mode, chlorin gold nanorods formulation increased Caspase3 expression in breast cancer cells^15^. Moreover, a previous study has shown that hollow gold nanospheres combined with PDT method induced the highest expression level of Caspase3 and mitochondria-related cell death in cancer cells^44^. Additionally, there are several reports supporting the activation of Bax expression via PDT strategy in different cancers, including cervical and breast tumors^45^. On the other hand, it should be stressed that on the last treatment day, no harmful effects due to the treatment procedure were detected in the mice's vital organs such as the liver and lungs, which is one of the drawbacks of some conventional therapies like the chemotherapy.

## Conclusion

To conclude, it is well established that triple-negative breast cancers that we have used as a model in our study are highly invasive due to their limited treatment options^46^. Therefore, our data supported the fact that the NC + PDT + PTT strategy might hold a promising substitute for chemotherapy to treat triple-negative breast cancers. These results led us to realize that there is a good possibility that the old and high risky treatment therapies might be replaced with this dual irradiation method, which demands further complementary studies.
